# Dual Effect of a Polymorphism in the Macrophage Migration Inhibitory Factor Gene Is Associated with New-Onset Graves Disease in a Taiwanese Chinese Population

**DOI:** 10.1371/journal.pone.0092849

**Published:** 2014-03-25

**Authors:** Yu-Huei Liu, Ching-Chu Chen, Chen-Ming Yang, Yi-Ju Chen, Fuu-Jen Tsai

**Affiliations:** 1 Department of Medical Genetics and Medical Research, China Medical University Hospital, Taichung, Taiwan; 2 Graduate Institute of Integrated Medicine, China Medical University, Taichung, Taiwan; 3 Division of Endocrinology and Metabolism, Department of Medicine, China Medical University Hospital, Taichung, Taiwan; 4 Department of Endocrinology and Metabolism, College of Chinese Medicine, China Medical University, Taichung, Taiwan; 5 School of Chinese Medicine, China Medical University, Taichung, Taiwan; 6 Department of Pediatrics, China Medical University Hospital, Taichung, Taiwan; 7 School of Post-Baccalaureate Chinese Medicine, China Medical University, Taichung, Taiwan; 8 Department of Biotechnology, Asia University, Taichung, Taiwan; 9 Department of Biotechnology and Bioinformatics, Asia University, Taichung, Taiwan; The Ohio State University, United States of America

## Abstract

Graves disease (GD) is an autoimmune disease. Macrophage migration inhibitory factor (MIF) is a potent cytokine that plays an important role in the regulation of immune responses. Two polymorphisms in the promoter region of *MIF*, rs5844572 and rs755622, are known to affect MIF expression. The purpose of this study was to investigate the relationship between polymorphisms in the *MIF* gene promoter and the severity of GD. A total of 677 individuals, including 481 GD patients and 196 ethnically matched healthy controls, were genotyped to identify differences in the distribution of the *MIF* polymorphisms rs5844572 and rs755622. Although there were no significant differences in the allele or genotype distributions among patients with different grades of goiter in GD and healthy controls, the distribution of the C allele, especially C/C genotype, of the rs755622 single nucleotide polymorphism (SNP) in *MIF*, may be as a risk factor for goiter initiation whereas a protector against development of severe goiter in patients with untreated GD (p<0.05). A goiter-developmental model incorporating genetic (*MIF* SNP rs755622) and environmental risk factors (gender, radioiodine treatment, thyroid gland surgery and vitiligo) significantly increased the prediction accuracy. Further studies are required to address the role of *MIF* polymorphisms, as well as their association with other candidate genes, in GD.

## Introduction

Graves disease (GD) is an autoimmune thyroid disease that occurs in approximately 5–12 per 1000 people in Western populations and in 3–11 per 1000 people in Chinese populations [1], [2]. The characteristics of GD include an enlarged thyroid gland, rapid heartbeat, and nervous excitability, with or without GD-related ophthalmopathy [2], [3]. Although the exact reason for autoantibody production remains uncertain, it appears to be caused by complex interactions between many genetic and environmental factors. Robust evidence from genome-wide association analyses has helped identify some of the GD-associated polymorphisms, such as those in the thyroid-stimulating hormone receptor (*TSHR*), thyroglobulin, human leukocyte antigen (*HLA*), cytotoxic T-lymphocyte antigen-4 (*CTLA-4*), and Fc receptor-like 3 (*FCRL3*) genes; however, the results of these studies also reiterate the complexity of the disease and the need for detection of additional genetic determinants of GD [4]–[11].

The macrophage migration inhibitory factor (*MIF*) gene, located on 22q11.2, encodes a multifunctional cytokine, MIF, which is produced by several types of cells, including epithelial cells and cells that participate in the innate and adaptive immune responses [12]–[14]. MIF is known to mediate certain cell-mediated immune responses, immune regulation, and inflammation. Overexpression and secretion of MIF help restore macrophage cytokine production and T cell activity in response to the immunosuppressive effects of glucocorticoids [15]. MIF deficiency causes abnormal secretion of mouse cytokines [16] and increases fibroblast adipogenesis in culture [17]. In addition, some studies suggest that MIF might be associated with alterations in thyroid hormone secretion in certain diseases [18]–[20]. Several studies have shown that two common functional polymorphisms of the *MIF* gene, rs5844572 (with 5–8 tetranucleotide CATT repeats at position –794 [21]) and rs755622 (with a G to C transition at position –173 [22]) are associated with susceptibility to or severity of several acute, chronic, and autoimmune inflammatory disorders [23]–[30]. However, only a few studies have been performed on the role of *MIF* polymorphisms in the development of GD. The aim of this study was to investigate the possible role of *MIF* polymorphisms in the development of GD.

## Materials and Methods

### Patients

A total of 481 GD patients and 196 ethnically matched healthy controls were enrolled in this study at the China Medical University Hospital in Taiwan. Detailed descriptions of the inclusion/exclusion criteria of GD have been published elsewhere as listed below [31]–[34]: the inclusion criteria including (a) patients are self-reported non-aboriginal Taiwaneses, and none of the parents and grand-parents has aboriginal background; (b) patients have to understand risks and benefits of the protocol and be able to give informed consent; (c) patients have typical clinical features including hyperthyroidism, diffuse enlargement of the thyroid gland, increased free thyroxine or triiodothyronine levels, suppressed thyroid stimulating hormone levels, positive thyrotrophin-receptor autoantibodies, and with or without antimicrosomal or antithyroglobulin antibodies; (d) patients have to satisfy the diagnostic criteria of GD at the time of examination; the exclusion criteria including (a) patients are unable to understand or give informed consent; (b) patients who had pregnancy or had delivered a baby/babies within one year. The definition of euthyroid patients were those with ranges for free thyroxine (FT4) of 0.54–1.40 ng/dL and thyroid stimulating hormone (TSH) of 0.34–5.60 mIU/L. All methods followed the tenets of the Declaration of Helsinki. All participants provided written informed consent and the Medical Ethics Committee of the China Medical University Hospital approved the study.

### Genomic DNA extraction and genotyping

Blood samples were collected by venipuncture and genomic DNA was extracted from peripheral blood leukocytes using a genomic DNA kit (Qiagen) in accordance with the manufacturer’s instructions. Genotyping methods were performed as described as follows:

For assessment of the rs5844572 (–794 CATT 5-8) polymorphism, the microsatellite was analyzed using polymerase chain reaction (PCR) followed by capillary electrophoresis with 20 ng of genomic DNA as the template in a 10-μL reaction volume using the method described in a previous report [35], with some modifications. In brief, the PCR was performed with 20 ng of genomic DNA as template in a 10-μL reaction using the following primers: forward primer, 5′-TTG CAC CTA TCA GAG ACC-3′ and reverse primer, 5′-TCC ACT AAT GGT AAA CTC G-3′; the primers were 5′-end labeled with 6-carboxyfluorescein (6-FAM). The PCR amplification was carried out in a 96-well thermal cycler (Bio-Rad) and the protocol comprised 40 cycles each of 95°C for 1 min, 54°C for 1 min, and 72°C for 45 s, followed by one cycle at 72°C for 7 min. Amplification was confirmed by 3% agarose gel electrophoresis before denaturation of the PCR products. One microliter of the PCR reaction was analyzed with an internal sizing standard (Applied Biosystems), on a DNA analyzer (Applied Biosystems). The polymorphism was identified using the Genemapper 4.0 software (Applied Biosystems) after capillary electrophoresis.

For assessment of the rs755622 (–173G/C) polymorphism, the genotyping was performed using the PCR-restriction fragment length polymorphism (PCR-RFLP) method described in a previous report [36], with some modifications. In brief, the PCR amplification was performed with 20 ng of genomic DNA as template in a 10-μL reaction using the following primers: forward primer, 5′-ACT AAG AAA GAC CCG AGG C-3′ and reverse primer, 5′-GGG GCA CGT TGG TGT TTA C-3′. The amplification was carried out in a 96-well thermal cycler (Bio-Rad) and the PCR protocol comprised 40 cycles each of 95°C for1 min, 54°C for 1 min, and 72°C for 1 min, followed by one cycle at 72°C for 7 min. The PCR products (268 bp and 97 bp) were digested with *Alu*I (New England Biolabs) at 37°C for 4 h followed by 3.5% agarose gel electrophoresis. Digestion of the PCR products with *Alu*I yielded 206-, 97-, and 62-bp fragments for the C allele and 268- and 97-bp fragments for the G allele.

### Statistical analyses

Sample size is estimated using A-priori analysis (power  =  0.8, α = 0.05). Statistical analyses were performed using the PASW Statistics 18.0 software package. Polymorphism frequencies among the groups were compared, and the odds ratios (ORs) with a 95% confidence interval (CI) were estimated by applying a logistic regression model. The factor–factor interaction models for severe goiter were detected by using the multifactor dimensionality reduction (MDR) 1.1.0 of the open-source MDR software package (Dartmouth Medical School, Hanover, NH). The interaction dendrogram was established according to a hierarchical clustering algorithm [37], [38].

## Results

### Allele and genotype distribution of *MIF* in GD patients and healthy controls

The demographic information and clinical characteristics of the 481 GD patients enrolled in this study are summarized in Table 1. The frequency of the polymorphisms examined was similar to those of the Chinese and Japanese (CHB and JPT) components of HapMap. No deviation from Hardy-Weinberg equilibrium was observed for allele frequencies of the rs5844572 and rs755622 polymorphisms in the *MIF* gene (P >0.05).

**Table 1:** Characteristics of patients with Graves disease.

		Graves disease, goiter grade					
Characteristic	Case number	0	1a	1b	2	3	P value
Female gender [n (%)]	381	28	22	43	242	46	0.513
	(79.2)	(87.5)	(84.6)	(79.6)	(79.1)	(73.0)	
Age [year, median (range)]	42.0	46.0	51.5	40.9	42.0	37.0	0.001
	(17–87)	(27–77)	(28–71)	(23–75)	(17–87)	(17–77)	
With cigarette smoking history [n (%)]	122	5	7	12	67	21	0.270
	(23.3)	(15.6)	(26.9)	(22.2)	(21.9)	(33.3)	
With radioiodine treatment [n (%)]	21	7	2	1	9	2	2.281×10^−5^
	(4.4)	(21.9)	(7.7)	(1.9)	(2.9)	(3.2)	
With thyroid gland surgery [n (%)]	48	20	1	3	21	3	5.887×10^−22^
	(10.0)	(62.5)	(3.8)	(5.6)	(6.9)	(4.8)	
With ophthalmopathy [n (%)]	202	14	5	22	131	30	0.164
	(42.0)	(43.8)	(19.2)	(40.7)	(42.8)	(47.6)	
With nodular hyperplasia [n (%)]	4	1	5	34	5	4	0.733
	(12.5)	(3.8)	(9.3)	(11.1)	(7.9)	(12.5)	
With myxedema [n (%)]	6	0	0	2	2	2	0.188
	(1.2)	(0.0)	(0.0)	(3.7)	(0.7)	(3.2)	
With vitiligo [n (%)]	4	0	2	1	1	0	0.002
	(0.8)	(0.0)	(7.7)	(1.9)	(0.3)	(0.0)	

The distribution of the *MIF* polymorphisms among patients with different severities of goiter (grades 0, 1A, 1B, 2, and 3) as well as in healthy controls were analyzed. A-priori analysis revealed that the minimum total sample size (two-tailed hypothesis) is 98 when consider the difference of GD patients with grade 0 goiter and other groups. The allele and genotype distributions of the rs5844572 and rs755622 polymorphisms in *MIF* were not significantly different among the patient and control groups (P >0.05; Table 2).

**Table 2:** Distributions of alleles and genotypes of the *MIF* polymorphisms among patients with Graves disease and healthy controls.

		Graves disease, goiter grade					
Polymorphisms, n (%)	Healthy	0	1a	1b	2	3	P value
rs5844572 -794(CATT)_n_							
5	142	23	15	39	200	41	0.988 a
	(36.2)	(35.9)	(28.8)	(36.1)	(32.7)	(32.5)	0.990 b
6	192	32	27	54	315	63	
	(49.0)	(50.0)	(51.9)	(50.0)	(51.5)	(50.0)	
7	54	9	10	15	92	21	
	(13.8)	(14.1)	(19.2)	(13.9)	(15.0)	(16.7)	
8	4	0	0	0	5	1	
	(1.0)	(0.0)	(0.0)	(0.0)	(0.8)	(0.8)	
5/5	19	2	2	9	29	5	0.968 a
	(9.7)	(6.3)	(7.7)	(16.7)	(9.5)	(7.9)	0.992 b
5/6	74	14	8	16	116	21	
	(37.8)	(43.8)	(30.8)	(29.6)	(37.9)	(33.3)	
5/7	26	5	3	5	25	9	
	(13.3)	(15.6)	(11.5)	(9.3)	(8.2)	(14.3)	
5/8	4	0	0	0	1	1	
	(2.0)	(0.0)	(0.0)	(0.0)	(0.3)	(1.6)	
6/6	48	7	7	15	74	17	
	(24.5)	(21.9)	(26.9)	(27.8)	(24.2)	(27.0)	
6/7	22	4	5	8	48	8	
	(11.2)	(12.5)	(19.2)	(14.8)	(15.7)	(12.7)	
6/8	0	0	0	0	3	0	
	(0.0)	(0.0)	(0.0)	(0.0)	(1.0)	(0.0)	
7/7	3	0	1	1	9	2	
	(1.5)	(0.0)	(3.8)	(1.9)	(2.9)	(3.2)	
7/8	0	0	0	0	1	0	
	(0.0)	(0.0)	(0.0)	(0.0)	(0.3)	(0.0)	
rs755622 G-173C							
G	76	17	10	13	90	20	0.068 a
	(19.4)	(26.6)	(19.2)	(12.0)	(14.7)	(15.9)	0.100 b
C	316	47	42	95	522	106	
	(80.6)	(73.4)	(80.8)	(88.0)	(85.3)	(84.1)	
G/G	128	19	17	43	222	44	0.079 a
	(65.3)	(59.4)	(65.4)	(79.6)	(72.5)	(69.8)	0.054 b
G/C	60	9	8	9	78	18	
	(30.6)	(28.1)	(30.8)	(16.7)	(25.5)	(28.6)	
C/C	8	4	1	2	6	1	
	(4.1)	(12.5)	(3.8)	(3.7)	(2.0)	(1.6)	

a Comparisons among healthy individuals and the five groups of different severity of goiter.

b Comparisons among the five groups of different severity of goiter.

### The C allele of the rs755622 SNP in *MIF* is associated with goiter severity in patients with untreated GD

Because medical therapies such as radioiodine treatment, thyroid gland surgery, and other drugs may reduce the severity of GD-associated phenotypes subsequently altering the polymorphism-related phenotype, we next examined the association of the polymorphisms with the severity of goiter using a stratified method of analysis, in which the patients were stratified based on whether the GD was euthyroid, untreated or treated. The demographic information and clinical characteristics of the subgroups are summarized in Table S1. It is notable that both the allele and genotype distribution of the rs755622 SNP in *MIF* showed associations with the severity of goiter in patients with untreated GD (GD patients vs healthy controls, P = 0.006 for allele distribution and P = 0.009 for genotype distribution; among GD patients with goiter of different grades, P = 0.002 for allele distribution and P = 0.001 for genotype distribution; Table 3). No association was found between the SNP and the severity of goiter in euthyroid group or the treated group (P > 0.05 for allele and genotype distribution; Table S2 and S3). A-priori analysis revealed that the minimum total sample size (two-tailed hypothesis) is 46 when consider the difference of untreated GD patients with grade 0 goiter and other groups. Therefore the comparison between GD patients with grade 0 goiter and healthy controls, as well as GD patients with grade 0 goiter and those with grade 2 goiter, were further analyzed. Logistic regression analyses revealed that in the untreated group, GD patients with the C allele may be risk for initial goiter development (odds ratio (OR): 5.821, 95% confidence interval (CI): 1.978–18.843 for GD patients with grade 0 goiter as compared to healthy individuals), but may be protected from severe goiter development (OR: 0.133, 95%CI: 0.040–0.438 for GD patients with goiter of grade 2, as compared to those with grade 0 goiter; Table 4). In addition, the C/C genotype may be risk for initial goiter development (OR: 32.000, 95%CI: 2.615–391.587 for GD patients with grade 0 goiter as compared to healthy individuals), but protective against development of severe goiter (OR: 0.016, 95%CI: 0.001–0.226 for GD patients with goiter of grades 1b, 2 and 3, respectively, as compared to those with grade 0 goiter; Table 4). The differences were statistically significant after adjustment for either age only, or age and gender, although the association was more pronounced in the female patients (Table S4). These results suggest that the C allele of the rs755622 SNP in *MIF*, especially the C/C genotype, may play a role as risk factor for goiter initiation, and may play a protective role against development of severe goiter in patients with untreated GD.

**Table 3:** Distributions of alleles and genotypes of the *MIF* polymorphisms with respect to the severity of goiter in patients with untreated Graves disease.

		Graves disease, goiter grade					
Polymorphisms, n (%)	Healthy	0	1a	1b	2	3	P value
rs5844572 -794(CATT)_n_							
5	142	4	5	16	84	22	0.979 a
	(36.2)	(33.3)	(31.3)	(36.4)	(31.3)	(39.3)	0.974 b
6	192	5	8	22	134	27	
	(49.0)	(41.7)	(50.0)	(50.0)	(50.0)	(48.2)	
7	54	3	3	6	47	6	
	(13.8)	(25.0)	(18.8)	(13.6)	(17.5)	(10.7)	
8	4	0	0	0	3	1	
	(1.0)	(0.0)	(0.0)	(0.0)	(1.1)	(1.8)	
5/5	19	1	0	3	12	4	0.935 a
	(9.7)	(16.7)	(0.0)	(13.6)	(9.0)	(14.3)	0.984 b
5/6	74	1	3	9	50	10	
	(37.8)	(16.7)	(37.5)	(40.9)	(37.3)	(35.7)	
5/7	26	1	2	1	9	3	
	(13.3)	(16.7)	(25.0)	(4.5)	(6.7)	(10.7)	
5/8	4	0	0	0	1	1	
	(2.0)	(0.0)	(0.0)	(0.0)	(0.7)	(3.6)	
6/6	48	1	2	4	29	8	
	(24.5)	(16.7)	(25.0)	(18.2)	(21.6)	(28.6)	
6/7	22	2	1	5	25	1	
	(11.2)	(33.3)	(12.5)	(22.7)	(18.7)	(3.6)	
6/8	0	0	0	0	1	0	
	(0.0)	(0.0)	(0.0)	(0.0)	(0.7)	(0.0)	
7/7	3	0	0	0	6	1	
	(1.5)	(0.0)	(0.0)	(0.0)	(4.5)	(3.6)	
7/8	0	0	0	0	1	0	
	(0.0)	(0.0)	(0.0)	(0.0)	(0.7)	(0.0)	
rs755622 G-173C							
G	76	7	4	7	42	7	0.006 a
	(19.4)	(58.3)	(25.0)	(15.9)	(15.7)	(12.5)	0.002 b
C	316	5	12	37	226	49	
	(80.6)	(41.7)	(75.0)	(84.1)	(84.3)	(87.5)	
G/G	128	1	4	16	95	21	0.009 a
	(65.3)	(16.7)	(50.0)	(72.7)	(70.9)	(75.0)	0.001 b
G/C	60	3	4	5	36	7	
	(30.6)	(50.0)	(50.0)	(22.7)	(26.9)	(25.0)	
C/C	8	2	0	1	3	0	
	(4.1)	(33.3)	(0.0)	(4.5)	(2.2)	(0.0)	

Abbreviations: MIF, macrophage migration inhibitory factor.

a Comparisons among healthy individuals and the five groups of different severity of goiter.

b Comparisons among the five groups of different severity of goiter.

**Table 4:** Odds ratios of severity of goiter cases categorized by *MIF* polymorphism rs755622 in patients with untreated Graves disease.

	Comparison to Healthy				Comparison to Grave disease, goiter grade 0			
Polymorphisms		OR	95%CI	P value		OR	95%CI	P value
G	H vs 0	1.000		0.001				
C		5.821	1.978–18.843					
G/G		1.000		0.002				
G/C		6.400	0.652–62.812					
C/C		32.000	2.615–391.587					
G	H vs 1a	1.000		0.579	0 vs 1a	1.000		0.074
C		1.386	0.435–4.417			0.238	0.048–1.193	
G/G		1.000		0.466		1.000		0.155
G/C		2.133	0.516–8.821			0.333	0.023–4.736	
C/C		0.000	0.000–			0.000	0.000–	
G	H vs 1b	1.000		0.577	0 vs 1b	1.000		0.003
C		0.787	0.338–1.833			0.135	0.033–0.550	
G/G		1.000		0.745		1.000		0.026
G/C		0.667	0.233–1.905			0.104	0.009–1.239	
C/C		1.000	0.117–8.524			0.031	0.001–0.720	
G	H vs 2	1.000		0.221	0 vs 2	1.000		1.417×10^−4^
C		0.773	0.511–1.169			0.133	0.040–0.438	
G/G		1.000		0.458		1.000		7.072×10^−5^
G/C		0.808	0.495–1.321			0.126	0.013–1.254	
C/C		0.505	0.131–1.955			0.016	0.001–0.226	
G	H vs 3	1.000		0.215	0 vs 3	1.000		3.661×10^−4^
C		0.594	0.259–1.363			0.102	0.025–0.411	
G/G		1.000		0.417		1.000		0.002
G/C		0.711	0.287–1.764			0.111	0.010–1.249	
C/C		0.000	0.000-			0.000	0.000-	

Abbreviations: MIF, macrophage migration inhibitory factor; OR, odd ratio; CI, confidence interval.

### The C allele of the rs755622 SNP in *MIF* and other clinical features of Graves disease

A comparison of clinical features [gender, age, frequency of radioiodine treatment, thyroid gland surgery, ophthalmopathy, nodular hyperplasia, myxedema, vitiligo, cigarette smoking habit, as well as thyroid functions including initial FT4, TSH and anti-thyroid hormone receptor antibody (TRAb) levels among rs755622 genotypes (G/G and G/C+C/C)] was shown. Results suggest that in the untreated group, the rs755622 SNP in *MIF* is also associated with age, thyroid gland surgery and vitiligo (Table 5). The results of euthyroid group and treated group are summarized in Table S5.

**Table 5:** Clinical significance of *MIF* genotype in patients with untreated Graves disease.

	rs755622 genotypes		
Characteristic	G/G (n = 137)	G/C + C/C (n = 61)	P value
Female gender [n (%)]	104	53	0.138
	(75.9)	(85.2)	
Age [year, median (range)]	37.0	42.0	0.027
	(17–77)	(22–74)	
With cigarette smoking history [n (%)]	37	15	0.721
	(71.2)	(28.8)	
With radioiodine treatment [n (%)]	3	2	0.652
	(2.2)	(3.3)	
With thyroid gland surgery [n (%)]	3	5	0.047
	(2.2)	(8.2)	
With ophthalmopathy [n (%)]	59	24	0.624
	(43.1)	(39.3)	
With nodular hyperplasia [n (%)]	8	6	0.311
	(5.8)	(9.8)	
With myxedema [n (%)]	0	1	0.133
	(0.0)	(1.6)	
With vitiligo [n (%)]	0	2	0.033
	(0.0)	(3.3)	
FT4 (ng/dL)[mean (SD)]	2.2	2.1	0.717
	(1.5)	(1.5)	
TSH (mIU/L)[mean (SD)]	2.9	2.5	0.857
	(13.8)	(14.0)	
TRAb (%)[mean (SD)]	52.0	49.0	0.588
	(24.3)	(23.6)	

Abbreviations: MIF, macrophage migration inhibitory factor; FT4, free thyroxine; TSH, thyroid stimulating hormone; TRAb, anti-thyroid hormone receptor antibody; SD, standard deviation.

The rs755622 SNP in *MIF* was shown no association toward any thyroid function in the untreated group (Table 5). However, an association has been found in the treated group: the patients carrying G/C or C/C genotypes at the rs755622 SNP in *MIF* were with higher FT4 level (2.28±1.52 ng/dL) as compared to those carrying G/G genotype (1.63±1.23 ng/dL) (p = 0.009; Table S5). The associations remained after the gender-stratified analyses: in male patients carrying G/C or C/C genotypes at the rs755622 SNP in *MIF* were with higher FT4 level (2.2±1.5 ng/dL) as compared to those carrying G/G genotype (1.3±1.0 ng/dL) (p = 0.029), and in female patients carrying G/C or C/C genotypes at the rs755622 SNP in *MIF* were with higher FT4 level (2.3±1.6 ng/dL) as compared to those carrying G/G genotype (1.6±1.3 ng/dL) (p = 0.030).

On the other hand, in patients with Graves disease, treated with radioactive iodine, the inhibition rate of the migration of leukocytes (indicates the production of MIF) appeared to be lower [39]. The results in this study demonstrated that higher percentage of patients carrying C/C genotype at the rs755622 SNP in *MIF* (3/14, 21.4%) have received radioiodine treatment as compared to those carrying G/C genotype (4/122, 3.3%) or G/G genotype (14/345, 4.1%) (p = 0.006). In the three patients carrying C/C genotype with radioiodine treatment, two of which were female (age 28 and age 33) and one is male (age 44). The observation might provide a crud when considering the role of MIF in the disease, but additional studies in larger cohorts are required.

### Multifactor dimensionality reduction analysis

**Figure 1:**
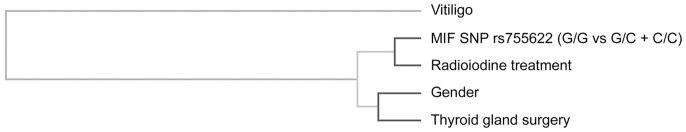
Interaction dedrogram. The location of the longitudinal connecting bars indicates the strength of the dependence: left is weaker and right is stronger. The hierarchical cluster analysis placed *MIF* SNP rs755622 genotype (GG vs GC+CC) and radioiodine treatment on the same branch, demonstrating the most strong interaction between these two factors. Gender, thyroid gland surgery and vitiligo are also included in the dendrogram as shown. *MIF*, macrophage migration inhibitory factor; SNP, single nucleotide polymorphism.

To extend the previous findings, the interaction between rs755622 SNP in *MIF* and other non-genetic factors on the severity of goiter (0 vs 1a/1b/2/3) in untreated GD patients was determined by using the MDR analysis. The results suggested that as compared to the one-factor model, rs755622 genotype of *MIF* (G/G, G/C + C/C) (testing balance accuracy: 77.08%, OR (95%CI): 12.143 (1.387–106.298), P = 0.0047), the 5-factor model consisted of the aforementioned genotype and the additional factors including gender, radioiodine treatment, thyroid gland surgery, and vitiligo showed to increase the prediction accuracy (testing balance accuracy: 86.72%, OR (95%CI): ∞, P<1.000×10^−4^). The interaction dendrogram was shown in Figure 1.

## Discussion

Recent studies in which genome-wide association analyses were performed in different populations have provided information about several common susceptibility loci for GD [4]–[11]. Genetic polymorphisms in *TSHR* are thought to be initiators in the development of GD [40], [41]. Polymorphisms in *HLA* may be associated with autoreactivity [42], whereas those in *CTLA*–*4* may be correlated with disturbance of T-cell suppression [43], [44]. Polymorphisms in *FCRL3* may be correlated with regulation of the immune system through its immunoreceptor-tyrosine activation and inhibitory motifs on the cytoplasmic domain [45], [46]. MIF is a multifunctional cytokine that is secreted from T lymphocytes, endothelial cells, macrophages, and other inflammatory cells [47]–[50]. Aberrant changes in MIF expression and function have been implicated or suggested in many inflammatory and immune response-related conditions, including rheumatoid arthritis [51], ankylosing spondylitis [52], and ocular inflammation [53]. To date, various thyrocyte, monocyte, macrophage, dendritic cell, B cell and T cell abnormalities have been found in the animal models of spontaneously developing autoimmune thyroiditis and in patients with autoimmune thyroid disease [54]–[56]. Given the apoptosis is one of the crucial mechanisms in the maintenance of cell population balance [55], [57], and the presence of high concentrations of MIF suppresses activated immune cells apoptosis through p53-dependent mechanism [58], *MIF* may play a role in priming these autoimmune disorders in the microenvironment. However, reports on the role of *MIF* polymorphisms and their effect on MIF expression in the development of GD are scarce. In this study, by using a candidate-based approach, we circumvented the limitations of methods based on genome-wide analyses, such as insufficient SNP coverage, difficulty with identifying genuine associations due to multiple testing corrections, and lack of detection of markers like microsatellites. We conclude from our data that genetic variation in the *MIF* gene may influence the severity of goiter in patients with untreated GD. To the best our knowledge, this is the first study to demonstrate that *MIF* polymorphisms may be associated with severity of goiter in GD.

Linkage between MIF production and thyroid antigens, including thyroid particulate antigen, thyroid microsomal antigen and long acting thyroid stimulator has been previously described in Graves disease [39]. The association between *MIF* polymorphisms and the thyroid levels have been analyzed, however, only an association has been found in the treated group, demonstrated that GD patients carrying G/C or C/C genotypes at the rs755622 SNP in *MIF* were with higher FT4 level compared to those carrying G/G genotype. In addition, attempts to show the association between *MIF* polymorphisms and TRAb levels have not been successful. One possible explanation is that there are other types of thyroid antigens and thyroid autoantibodies that should be considered. Moreover, the higher percentage in patients carrying C/C genotype at the rs755622 SNP in *MIF* have received radioiodine treatment might provide another view for considering the role of MIF in the disease. The detail mechanism seems not easily understood which required further investigation.

On the other hand, when thinking the implications of these genetic findings to patients with Hashimoto's thyroiditis, another thyroid autoimmune disease, yet no related evidence has been reported, although the production of MIF is detectable in both diseases. Since the original role of MIF is linked to the cell mediated immunity-induced tissue destructive lesions that has been accepted to play a role in Hashimoto’s thyroiditis [39], another interest will be addressed on the similarity and the difference of MIF in both diseases.

Based on this genetic study, we cannot ascribe causality to any particular *MIF* allele. However, reports have described the association between high-expression *MIF* alleles and increased MIF production, showing that the C allele of the rs755622 introduces an activator protein 4 (AP-4) transcription factor binding site [21], [26], [29], [30], [59]. It appears likely that a robust MIF response plays a protective role against development of severe goiter in patients with untreated GD. One reason for the specific effect of *MIF* in untreated populations may be the involvement of different pathogenic mechanisms (e.g., antagonization of MIF-related effects by thyroxine [19], [20]) at different stages of GD. In addition, these observations point to the possibility that the patients with ongoing or recurrent forms of GD might have already developed a steady programmed cellular response through other regulatory mechanisms (e.g., epigenetic regulation), which might diminish the effect of *MIF* polymorphisms on MIF production and/or secretion at later stages of the disease. Moreover, the possibility that both cell type-specific and time-course-of-disease-dependent changes in the transcription of *MIF* might affect its role in the prevention of severe goiter cannot be excluded [22], [60]. A similar example for dual effect of *MIF* polymorphism is observed in another autoimmune disease, systemic lupus erythematosus [61]. Further studies in larger cohorts and a comprehensive analysis of the clinicopathological characteristics, including gender difference, of GD patients are required to address the role of MIF and its association with other candidate GD-associated genes. In addition, other ethnic populations need to be included to confirm and generalize the current conclusion.

The mechanism through which MIF functions in both the extra- and intracellular spaces remains controversial. MIF has been implicated in the pathogenesis of multiple systemic and organ-specific autoimmune diseases [23]–[30] because it promotes the activation and secretion of proinflammatory cytokines by immune cells [15], [62]. In GD, the overproduction of MIF may potentiate inflammation and priming of naive autoreactive T (major Th1) cells, thereby enhancing the adverse effects on other organs. On the other hand, MIF has been recognized as a “watchman”, which protects the host from immune responses capable of overtaxing the hosts systems [63], [64]. The specific function of MIF that is affected in GD and the mechanism(s) underlying the association of the rs755622 SNP in MIF with goiter in GD need to be addressed in further studies.

In summary, our findings provide new information pertaining to the role of *MIF* gene polymorphisms and show that the rs755622 SNP is associated with the severity of goiter in patients with untreated GD in a Taiwanese Chinese population. These data suggest the need for additional studies in larger cohorts to address the role of *MIF* polymorphisms in the pathogenesis of GD as well as their relationship with other candidate genes with known/putative functions in GD.

## Supporting Information

Table S1 **Characteristics of in the subgroups of patients with Graves disease.**
(DOCX)

Table S2 **Distributions of alleles and genotypes of the** ***MIF*** **polymorphisms with respect to the severity of goiter in patients with euthyroid Graves disease.**
(DOCX)

Table S3 **Distributions of alleles and genotypes of the** ***MIF*** **polymorphisms with respect to the severity of goiter in patients with treated Graves disease.**
(DOCX)

Table S4 **Gender effects on distributions of alleles and genotypes of the** ***MIF*** **polymorphisms with respect to the severity of goiter in patients with untreated Graves disease.**
(DOCX)

Table S5 **Clinical Significance of MIF Genotype in patients with euthyroid and untreated Graves disease.**
(DOCX)
