# Supplementary material for: Dual Effect of a Polymorphism in the Macrophage Migration Inhibitory Factor Gene Is Associated with New-Onset Graves Disease in a Taiwanese Chinese Population
Source: PLoS One. 2014 Mar 25;9(3):e92849. doi: 10.1371/journal.pone.0092849 (PMC3965479; doi:10.1371/journal.pone.0092849)
Supplement: Table S1 — Characteristics of in the subgroups of patients with Graves disease. (DOCX) [file pone.0092849.s001.docx]

Table S1. Characteristics of in the subgroups of patients with Graves disease.

|  | Graves disease, euthyroid | | | | |  | Graves disease, untreated group | | | | |  | Graves disease, treated group | | | | |  |
| --- | --- | --- | --- | --- | --- | --- | --- | --- | --- | --- | --- | --- | --- | --- | --- | --- | --- | --- |
|  | Goiter grade | | | | |  | Goiter grade | | | | |  | Goiter grade | | | | |  |
| Characteristic | 0 | 1a | 1b | 2 | 3 | P value | 0 | 1a | 1b | 2 | 3 | P value | 0 | 1a | 1b | 2 | 3 | P value |
| Female gender [*n* (%)] | 7 | 11 | 20 | 55 | 11 | 0.410 | 5 | 7 | 16 | 108 | 20 | 0.719 | 16 | 4 | 7 | 79 | 15 | 0.696 |
|  | (100.0) | (100.0) | (87.0) | (83.3) | (78.6) |  | (83.3) | (87.5) | (72.7) | (80.6) | (71.4) |  | (84.2) | (57.1) | (77.8) | (74.5) | (71.4) |  |
| Age [year, median (range)] | 39.0 | 53.0 | 43.0 | 40.9 | 41.0 | 0.020 | 40.0 | 49.5 | 37.4 | 39.0 | 32.5 | 0.162 | 50.0 | 43 | 43 | 46.9 | 38 | 0.298 |
|  | (35-65) | (37-71) | (23-75) | (20-74) | (21-60) |  | (29-72) | (28-55) | (24-71) | (17-74) | (17-77) |  | (27-77) | (28-60) | (29-59) | (24-87) | (27-71) |  |
| With cigarette smoking history [*n* (%)] | 1 | 1 | 3 | 13 | 1 | 0.723 | 2 | 1 | 6 | 30 | 13 | 0.097 | 2 | 5 | 3 | 24 | 7 | 0.022 |
|  | (14.3) | (9.1) | (13.0) | (19.7) | (7.1) |  | (33.3) | (12.5) | (27.3) | (22.4) | (46.4) |  | (10.5) | (71.4) | (33.3) | (22.6) | (33.3) |  |
| With radioiodine treatment [*n* (%)] | 0 | 0 | 1 | 0 | 1 | 0.284 | 2 | 0 | 0 | 2 | 1 | 6.004 × 10^-5^ | 5 | 2 | 0 | 7 | 0 | 0.006 |
|  | (0.0) | (0.0) | (4.3) | (0.0) | (7.1) |  | (33.3) | (0.0) | (0.0) | (1.5) | (3.6) |  | (26.3) | (28.6) | (0.0) | (6.6) | (0.0) |  |
| With thyroid gland surgery [*n* (%)] | 6 | 0 | 1 | 3 | 1 | 7.856 × 10^-11^ | 3 | 0 | 1 | 4 | 0 | 5.604 × 10^-7^ | 11 | 1 | 1 | 14 | 2 | 9.541 × 10^-5^ |
|  | (85.7) | (0.0) | (4.3) | (4.5) | (7.1) |  | (50.0) | (0.0) | (4.5) | (3.0) | (0.0) |  | (57.9) | (14.3) | (11.1) | (13.2) | (9.5) |  |
| With ophthalmopathy [*n* (%)] | 3 | 0 | 9 | 25 | 6 | 0.144 | 4 | 2 | 10 | 56 | 11 | 0.619 | 7 | 3 | 3 | 50 | 13 | 0.496 |
|  | (42.9) | (0.0) | (39.1) | (37.9) | (42.9) |  | (66.7) | (25.0) | (45.5) | (41.8) | (39.3) |  | (36.8) | (42.9) | (33.3) | (47.2) | (61.9) |  |
| With nodular hyperplasia [*n* (%)] | 1 | 1 | 3 | 6 | 0 | 0.724 | 0 | 0 | 0 | 11 | 3 | 0.467 | 3 | 0 | 2 | 17 | 2 | 0.696 |
|  | (14.3) | (9.1) | (13.0) | (9.1) | (0.0) |  | (0.0) | (0.0) | (0.0) | (8.2) | (10.7) |  | (15.8) | (0.0) | (22.2) | (16.0) | (9.5) |  |
| With myxedema [*n* (%)] | 0 | 0 | 1 | 1 | 0 | 0.814 | 0 | 0 | 1 | 0 | 1 | 0.192 | 0 | 0 | 0 | 1 | 1 | 0.617 |
|  | (0.0) | (0.0) | (4.3) | (1.5) | (0.0) |  | (0.0) | (0.0) | (4.5) | (0.0) | (3.6) |  | (0.0) | (0.0) | (0.0) | (0.9) | (4.8) |  |
| With vitiligo [*n* (%)] | 0 | 1 | 1 | 0 | 0 | 0.182 | 0 | 1 | 0 | 0 | 0 | 8.479 × 10^-5^ | 0 | 0 | 0 | 1 | 0 | 0.970 |
|  | (0.0) | (9.1) | (4.3) | (0.0) | (0.0) |  | (0.0) | (12.5) | (0.0) | (0.0) | (0.0) |  | (0.0) | (0.0) | (0.0) | (0.9) | (0.0) |  |
