# Supplementary material for: Dual Effect of a Polymorphism in the Macrophage Migration Inhibitory Factor Gene Is Associated with New-Onset Graves Disease in a Taiwanese Chinese Population
Source: PLoS One. 2014 Mar 25;9(3):e92849. doi: 10.1371/journal.pone.0092849 (PMC3965479; doi:10.1371/journal.pone.0092849)
Supplement: Table S2 — Distributions of alleles and genotypes of the MIF polymorphisms with respect to the severity of goiter in patients with euthyroid Graves disease. (DOCX) [file pone.0092849.s002.docx]

Table S2. Distributions of alleles and genotypes of the *MIF* polymorphisms with respect to the severity of goiter in patients with euthyroid Graves disease.

| Genotype |  | Graves disease, goiter grade | | | | |  |
| --- | --- | --- | --- | --- | --- | --- | --- |
| Polymorphisms, n (%) | Healthy | 0 | 1a | 1b | 2 | 3 | P value |
| rs5844572 -794(CATT)_n_ |  |  |  |  |  |  |  |
| 5 | 142 | 6 | 6 | 19 | 42 | 8 | 0.979 ^a^ |
|  | (36.2) | (42.9) | (27.3) | (41.3) | (31.8) | (28.6) | 0.958 ^b^ |
| 6 | 192 | 6 | 13 | 23 | 66 | 15 |  |
|  | (49.0 | (42.9) | (59.1) | (50.0) | (50.0) | (53.6) |  |
| 7 | 54 | 2 | 3 | 4 | 23 | 5 |  |
|  | (13.8 | (14.3) | (13.6) | (8.7) | (17.4) | (17.9) |  |
| 8 | 4 | 0 | 0 | 0 | 1 | 0 |  |
|  | (1.0 | (0.0) | (0.0) | (0.0) | (0.8) | (0.0) |  |
| 5/5 | 19 | 0 | 1 | 6 | 4 | 1 | 0.862 ^a^ |
|  | (9.7) | (0.0) | (9.1) | (26.1) | (6.1) | (7.1) | 0.724 ^b^ |
| 5/6 | 74 | 5 | 3 | 5 | 24 | 4 |  |
|  | (37.8) | (71.4) | (27.3) | (21.7) | (36.4) | (28.6) |  |
| 5/7 | 26 | 1 | 1 | 2 | 10 | 2 |  |
|  | (13.3) | (14.3) | (9.1) | (8.7) | (15.2) | (14.3) |  |
| 5/8 | 4 | 0 | 0 | 0 | 0 | 0 |  |
|  | (2.0) | (0.0) | (0.0) | (0.0) | (0.0) | (0.0) |  |
| 6/6 | 48 | 0 | 4 | 8 | 16 | 4 |  |
|  | (24.5) | (0.0) | (36.4) | (34.8) | (24.2) | (28.6) |  |
| 6/7 | 22 | 1 | 2 | 2 | 9 | 3 |  |
|  | (11.2) | (14.3) | (18.2) | (8.7) | (13.6) | (21.4) |  |
| 6/8 | 0 | 0 | 0 | 0 | 1 | 0 |  |
|  | (0.0) | (0.0) | (0.0) | (0.0) | (1.5) | (0.0) |  |
| 7/7 | 3 | 0 | 0 | 0 | 2 | 0 |  |
|  | (1.5) | (0.0) | (0.0) | (0.0) | (3.0) | (0.0) |  |
| 7/8 | 0 | 0 | 0 | 0 | 0 | 0 |  |
|  | (0.0) | (0.0) | (0.0) | (0.0) | (0.0) | (0.0) |  |
| rs755622 G-173C |  |  |  |  |  |  |  |
| G | 316 | 11 | 19 | 42 | 111 | 23 | 0.548 ^a^ |
|  | (80.6) | (78.6) | (86.4) | (91.3) | (84.1) | (82.1) | 0.700 ^b^ |
| C | 76 | 3 | 3 | 4 | 21 | 5 |  |
|  | (19.4) | (21.4) | (13.6) | (8.7) | (15.9) | (17.9) |  |
| G/G | 128 | 5 | 8 | 19 | 47 | 10 | 0.707 ^a^ |
|  | (65.3) | (71.4) | (72.7) | (82.6) | (71.2) | (71.4) | 0.704 ^b^ |
| G/C | 60 | 1 | 3 | 4 | 17 | 3 |  |
|  | (30.6) | (14.3) | (27.3) | (17.4) | (25.8) | (21.4) |  |
| C/C | 8 | 1 | 0 | 0 | 2 | 1 |  |
|  | (4.1) | (14.3) | (0.0) | (0.0) | (3.0) | (7.1) |  |

^a^ Comparisons among healthy individuals and the five groups of different severity of goiter.

^b^ Comparisons among the five groups of different severity of goiter.
