# Supplementary material for: Dual Effect of a Polymorphism in the Macrophage Migration Inhibitory Factor Gene Is Associated with New-Onset Graves Disease in a Taiwanese Chinese Population
Source: PLoS One. 2014 Mar 25;9(3):e92849. doi: 10.1371/journal.pone.0092849 (PMC3965479; doi:10.1371/journal.pone.0092849)
Supplement: Table S3 — Distributions of alleles and genotypes of the MIF polymorphisms with respect to the severity of goiter in patients with treated Graves disease. (DOCX) [file pone.0092849.s003.docx]

Table S3. Distributions of alleles and genotypes of the *MIF* polymorphisms with respect to the severity of goiter in patients with treated Graves disease.

| Genotype |  | Graves disease, goiter grade | | | | |  |
| --- | --- | --- | --- | --- | --- | --- | --- |
| Polymorphisms, n (%) | Healthy | 0 | 1a | 1b | 2 | 3 | P value |
| rs5844572 -794(CATT)_n_ |  |  |  |  |  |  |  |
| 5 | 142 | 13 | 4 | 4 | 74 | 11 | 0.481 ^a^ |
|  | (36.2) | (34.2) | (28.6) | (22.2) | (34.9) | 26.2 | 0.414 ^b^ |
| 6 | 192 | 21 | 6 | 9 | 115 | 21 |  |
|  | (49.0) | (55.3) | (42.9) | (50.0) | (54.2) | 50.0 |  |
| 7 | 54 | 4 | 4 | 5 | 22 | 10 |  |
|  | (13.8) | (10.5) | (28.6) | (27.8) | (10.4) | 23.8 |  |
| 8 | 4 | 0 | 0 | 0 | 1 | 0 |  |
|  | (1.0) | (0.0) | (0.0) | (0.0) | (0.5) | 0.0 |  |
| 5/5 | 19 | 1 | 1 | 0 | 13 | 0 | 0.548 ^a^ |
|  | (9.7) | (5.3) | (14.3) | (0.0) | (12.3) | (0.0) | 0.421 ^b^ |
| 5/6 | 74 | 8 | 2 | 2 | 42 | 7 |  |
|  | (37.8) | (42.1) | (28.6) | (22.2) | (39.6) | (33.3) |  |
| 5/7 | 26 | 3 | 0 | 2 | 6 | 4 |  |
|  | (13.3) | (15.8) | (0.0) | (22.2) | (5.7) | (19.0) |  |
| 5/8 | 4 | 0 | 0 | 0 | 0 | 0 |  |
|  | (2.0) | (0.0) | (0.0) | (0.0) | (0.0) | (0.0) |  |
| 6/6 | 48 | 6 | 1 | 3 | 29 | 5 |  |
|  | (24.5) | (31.6) | (14.3) | (33.3) | (27.4) | (23.8) |  |
| 6/7 | 22 | 1 | 2 | 1 | 14 | 4 |  |
|  | (11.2) | (5.3) | (28.6) | (11.1) | (13.20 | (19.0) |  |
| 6/8 | 0 | 0 | 0 | 0 | 1 | 0 |  |
|  | (0.0) | (0.0) | (0.0) | (0.0) | (0.9) | (0.0) |  |
| 7/7 | 3 | 0 | 1 | 1 | 1 | 1 |  |
|  | (1.5) | (0.0) | (14.3) | (11.1) | (0.9) | (4.8) |  |
| 7/8 | 0 | 0 | 0 | 0 | 0 | 0 |  |
|  | (0.0) | (0.0) | (0.0) | (0.0) | (0.0) | (0.0) |  |
| rs755622 G-173C |  |  |  |  |  |  |  |
| G | 316 | 31 | 11 | 16 | 185 | 34 | 0.407 ^a^ |
|  | (80.6) | (81.6) | (78.6) | (88.9) | (87.3) | (81.0) | 0.660 ^b^ |
| C | 76 | 7 | 3 | 2 | 27 | 8 |  |
|  | (19.4) | (18.4) | (21.4) | (11.1) | (12.7) | (19.0) |  |
| G/G | 128 | 13 | 5 | 8 | 80 | 13 | 0.177 ^a^ |
|  | (65.3) | (68.4) | (71.4) | (88.9) | (75.5) | (61.9) | 0.087 ^b^ |
| G/C | 60 | 5 | 1 | 0 | 25 | 8 |  |
|  | (30.6) | (26.3) | (14.3) | (0.0) | (23.6) | (38.1) |  |
| C/C | 8 | 1 | 1 | 1 | 1 | 0 |  |
|  | (4.1) | (5.3) | (14.3) | (11.1) | (0.9) | (0.0) |  |

^a^ Comparisons among healthy individuals and the five groups of different severity of goiter.

^b^ Comparisons among the five groups of different severity of goiter.
