# Supplementary material for: Dual Effect of a Polymorphism in the Macrophage Migration Inhibitory Factor Gene Is Associated with New-Onset Graves Disease in a Taiwanese Chinese Population
Source: PLoS One. 2014 Mar 25;9(3):e92849. doi: 10.1371/journal.pone.0092849 (PMC3965479; doi:10.1371/journal.pone.0092849)
Supplement: Table S4 — Gender effects on distributions of alleles and genotypes of the MIF polymorphisms with respect to the severity of goiter in patients with untreated Graves disease. (DOCX) [file pone.0092849.s004.docx]

Table S4. Gender effects on distributions of alleles and genotypes of the *MIF* polymorphisms with respect to the severity of goiter in patients with untreated Graves disease.

|  |  | Graves disease, goiter grade | | | | |  |
| --- | --- | --- | --- | --- | --- | --- | --- |
| Polymorphisms, n (%) | Healthy | 0 | 1a | 1b | 2 | 3 | P value |
| Male |  |  |  |  |  |  |  |
| G | 41 | 1 | 0 | 2 | 5 | 2 | 0.110 ^a^ |
|  | (26.3) | (50.0) | (0.0) | (16.7) | (9.6) | (12.5) | 0.468 ^b^ |
| C | 115 | 1 | 2 | 10 | 47 | 14 |  |
|  | (73.7) | (50.0 | (100.0) | (83.3) | (90.4) | (87.5) |  |
| G/G | 42 | 0 | 1 | 5 | 21 | 6 | 0.172 ^a^ |
|  | (53.8) | (0.0) | (100.0) | (83.3) | (80.8) | (75.0) | 0.160 ^b^ |
| G/C | 31 | 1 | 0 | 0 | 5 | 2 |  |
|  | (39.7) | (100.0) | (0.0) | (0.0) | (19.2) | (25.0) |  |
| C/C | 5 | 0 | 0 | 1 | 0 | 0 |  |
|  | (6.4) | (0.0) | (0.0) | (16.7) | (0.0) | (0.0) |  |
| Total | 78 | 1 | 1 | 6 | 26 | 8 |  |
|  | (100.0) | (100.0) | (100.0) | (100.0) | (100.0) | (100.0) |  |
| Female |  |  |  |  |  |  |  |
| G | 35 | 6 | 4 | 5 | 37 | 5 | 0.007 ^a^ |
|  | (14.8) | (60.0) | (28.6) | (15.6) | (17.1) | (12.5) | 0.008 ^b^ |
| C | 201 | 4 | 10 | 27 | 179 | 35 |  |
|  | (85.2) | (40.0) | (71.4) | (84.4) | (82.9) | (87.5) |  |
| G/G | 86 | 1 | 3 | 11 | 74 | 15 | 6.608 × 10^-4 a^ |
|  | (72.9) | (20.0) | (42.9) | (68.8) | (68.5) | (75.0) | 0.001 ^b^ |
| G/C | 29 | 2 | 4 | 5 | 31 | 5 |  |
|  | (24.6) | (40.0) | (57.1) | (31.3) | (28.7) | (25.0) |  |
| C/C | 3 | 2 | 0 | 0 | 3 | 0 |  |
|  | (2.5) | (40.0) | (0.0) | (0.0) | (2.8) | (0.0) |  |
| Total | 118 | 5 | 7 | 16 | 108 | 20 |  |
|  | (100.0) | (100.0) | (100.0) | (100.0) | (100.0) | (100.0) |  |

^a^ Comparisons among healthy individuals and the five groups of different severity of goiter.

^b^ Comparisons among the five groups of different severity of goiter.
