# Supplementary material for: Dual Effect of a Polymorphism in the Macrophage Migration Inhibitory Factor Gene Is Associated with New-Onset Graves Disease in a Taiwanese Chinese Population
Source: PLoS One. 2014 Mar 25;9(3):e92849. doi: 10.1371/journal.pone.0092849 (PMC3965479; doi:10.1371/journal.pone.0092849)
Supplement: Table S5 — Clinical Significance of MIF Genotype in patients with euthyroid and untreated Graves disease. (DOCX) [file pone.0092849.s005.docx]

Table S5. Clinical Significance of MIF Genotype in patients with euthyroid and untreated Graves disease.

|  | Euthyroid | |  |  | Untreated | |  |
| --- | --- | --- | --- | --- | --- | --- | --- |
|  | rs755622 genotypes | |  |  | rs755622 genotypes | |  |
| Characteristic | G/G  (n = 89) | G/C + C/C  (n = 32) | P value |  | G/G  (n = 119) | G/C + C/C  (n = 43) | P value |
| Female gender [n (%)] | 76 | 28 | 0.769 |  | 91 | 30 | 0.386 |
|  | (85.4) | (87.5) |  |  | (76.5) | (69.8) |  |
| Age [year, median (range)] | 42.0 | 43.9 | 0.024 |  | 46.9 | 42.5 | 0.065 |
|  | (20-75) | (23-70) |  |  | (27-87) | (24-69) |  |
| With cigarette smoking history [n (%)] | 37 | 15 | 0.721 |  | 31 | 10 | 0.718 |
|  | (71.2) | (28.8) |  |  | (26.1) | (23.3) |  |
| With radioiodine treatment [n (%)] | 1 | 1 | 0.446 |  | 10 | 4 | 0.857 |
|  | (1.1) | (3.1) |  |  | (8.4) | (9.3) |  |
| With thyroid gland surgery [n (%)] | 9 | 2 | 0.515 |  | 20 | 9 | 0.546 |
|  | (10.1) | (6.3) |  |  | (16.8) | (20.9) |  |
| With ophthalmopathy [n (%)] | 33 | 10 | 0.555 |  | 57 | 19 | 0.676 |
|  | (37.1) | (31.3) |  |  | (47.9) | (44.2) |  |
| With nodular hyperplasia [n (%)] | 7 | 4 | 0.434 |  | 19 | 5 | 0.492 |
|  | (7.9) | (12.5) |  |  | (16.0) | (11.6) |  |
| With myxedema [n (%)] | 1 | 1 | 0.446 |  | 1 | 0 | 0.547 |
|  | (1.10) | (3.1) |  |  | (0.8) | (0.0) |  |
| With vitiligo [n (%)] | 2 | 0 | 0.392 |  | 2 | 0 | 0.392 |
|  | (2.2) | (0.0) |  |  | (1.7) | (0.0) |  |
| FT4 (ng/dL)[mean (SD)] | 0.9 | 0.9 | 0.676 |  | 1.6 | 2.3 | 0.009 |
|  | (0.2) | (0.2) |  |  | (1.2) | (1.5) |  |
| TSH (mIU/L)[mean (SD)] | 2.1 | 2.3 | 0.524 |  | 2.9 | 4.3 | 0.522 |
|  | (1.4) | (1.30) |  |  | (9.8) | (16.5) |  |
| TRAb (%)[mean (SD)] | 30.9 | 30.8 | 0.977 |  | 41.7 | 43.3 | 0.765 |
|  | (20.6) | (25.1) |  |  | (26.5) | (31.0) |  |
